# Impact of oral anticoagulation on the association between frailty and clinical outcomes in people with atrial fibrillation: nationwide primary care records on treatment analysis

**DOI:** 10.1093/europace/euac022

**Published:** 2022-03-04

**Authors:** Chris Wilkinson, Jianhua Wu, Andrew Clegg, Ramesh Nadarajah, Kenneth Rockwood, Oliver Todd, Chris P Gale

**Affiliations:** Population Health Sciences Institute, Faculty of Medical Sciences, Newcastle University, Newcastle upon Tyne, UK; Leeds Institute of Cardiovascular and Metabolic Medicine, University of Leeds, 6.090a Worsley Building, Leeds LS2 9JT, UK; Leeds Institute for Data Analytics, University of Leeds, 6.090a Worsley Building, Leeds LS2 9JT, UK; Academic Unit for Ageing and Stroke Research, Leeds Institute of Health Sciences, University of Leeds, 6.090a Worsley Building, Leeds LS2 9JT, UK; Bradford Institute for Health Research, Bradford Teaching Hospitals NHS Foundation Trust, Bradford, UK; Leeds Institute of Cardiovascular and Metabolic Medicine, University of Leeds, 6.090a Worsley Building, Leeds LS2 9JT, UK; Leeds Institute for Data Analytics, University of Leeds, 6.090a Worsley Building, Leeds LS2 9JT, UK; Geriatric Medicine, Dalhousie University, Halifax, Nova Scotia, Canada; Academic Unit for Ageing and Stroke Research, Leeds Institute of Health Sciences, University of Leeds, 6.090a Worsley Building, Leeds LS2 9JT, UK; Bradford Institute for Health Research, Bradford Teaching Hospitals NHS Foundation Trust, Bradford, UK; Leeds Institute of Cardiovascular and Metabolic Medicine, University of Leeds, 6.090a Worsley Building, Leeds LS2 9JT, UK; Leeds Institute for Data Analytics, University of Leeds, 6.090a Worsley Building, Leeds LS2 9JT, UK; Department of Cardiology, Leeds Teaching Hospitals NHS Trust, Leeds, UK

**Keywords:** Frailty, Atrial fibrillation, Oral anticoagulation, Stroke, Bleeding, Oral anticoagulation prescription, Outcome

## Abstract

**Aims:**

People with atrial fibrillation (AF) frequently live with frailty, which increases the risk of mortality and stroke. This study reports the association between oral anticoagulation (OAC) and outcomes for people with frailty, and whether there is overall net benefit from treatment in people with AF.

**Methods and results:**

Retrospective open cohort electronic records study. Frailty was identified using the electronic frailty index. Primary care electronic health records of 89 996 adults with AF and CHA_2_DS_2_-Vasc score of ≥2 were linked with secondary care and mortality data in the Clinical Practice Research Database (CPRD) from 1 January 1998 to 30 November 2018. The primary outcome was a composite of death, stroke, systemic embolism, or major bleeding. Secondary outcomes were stroke, major bleeding, all-cause mortality, transient ischaemic attack, and falls. Of 89 996 participants, 71 256 (79.2%) were living with frailty. The prescription of OAC increased with degree of frailty. For patients not prescribed OAC, rates of the primary outcome increased alongside frailty category. Prescription of OAC was associated with a reduction in the primary outcome for each frailty category [adjusted hazard ratio, 95% confidence interval, no OAC as reference; fit: vitamin K antagonist (VKA) 0.69, 0.64–0.75, direct oral anticoagulant (DOAC) 0.42, 0.33–0.53; mild frailty: VKA 0.52, 0.50–0.54, DOAC 0.57, 0.52–0.63; moderate: VKA 0.54, 0.52–0.56, DOAC 0.57, 0.52–0.63; severe: VKA 0.48, 0.45–0.51, DOAC 0.58, 0.52–0.65], with cumulative incidence function effects greater for DOAC than VKA.

**Conclusion:**

Frailty among people with AF is common. The OAC was associated with a reduction in the primary endpoint across all degrees of frailty.

What’s new?

- In the absence of trial evidence, carefully conducted observational data have an important role. To our knowledge, this is the first study to use primary care electronic health records linked to hospital and mortality data to study the on-treatment effects of OAC on clinical outcomes among people with AF according to frailty status. The study demonstrates that prescription of OAC is associated with a reduction in the composite endpoint of death, stroke, systemic embolism, or major bleeding across the frailty spectrum.
- The finding that OAC prescription is associated with net clinical benefit across the frailty spectrum in people with AF is of importance to a large and growing population.

## Introduction

Atrial fibrillation (AF) is a major risk factor for thromboembolic stroke, which causes substantial morbidity and mortality.^1^ Every year in Europe around 800 000 strokes are considered to be related to AF.^2^ Although the risk of AF-related stroke is substantially reduced by oral anticoagulation (OAC),^1^ the evidence to guide the treatment of people with AF and concomitant frailty is less clear.

Frailty describes a state of vulnerability to adverse outcomes due to failure of homeostatic mechanisms and a reduction in physiological reserves.^3^ It is common in older people with AF, and is considered useful in guiding individualized treatment of people with cardiovascular disease.^4–6^ In those living with frailty, the balance of risk and benefit associated with OAC may be complex,^7^ yet the 2020 European Society of Cardiology Clinical Practice Guideline state that: ‘Frailty, comorbidities, and increased risk of falls do not outweigh the benefits of OAC given the small absolute risk of bleeding in anticoagulated elderly patients’.^8^ This statement is not supported by reference to outcomes data for patients with frailty and there is a gap in the evidence concerning the association between frailty and clinical outcomes by OAC prescription for people with AF who are at higher risk of stroke.

To address this, we undertook an open cohort study of primary care data for 89 996 patients with AF, linked to hospital records and national mortality data to quantify rates of all-cause mortality, stroke, severe bleeding, transient ischaemic attack (TIA), and falls; and examined associations between frailty and OAC prescription for these outcomes.

## Methods

### Setting and participants

We used electronic health records (EHR) data from the Clinical Practice Research Datalink (CPRD) Gold, which includes data from over 19 million patients registered at 394 general practices across the UK.^9^ Records were linked by CPRD to hospital admissions data from Hospital Episode Statistics (HES), cause of death data from the UK Office for National Statistics, and to local measures of deprivation [indices of multiple deprivation (IMD) and Townsend score]. Clinical diagnoses were identified using ICD-10 and Read codes (Supplementary material online, *Appendix S1*), which have been shown to have high reported accuracy in UK EHR.^10^

Participants were included in the study if they were aged 18 years or older, received a new diagnosis of non-valvular AF (paroxysmal, persistent, or permanent) or atrial flutter, and their CHA_2_DS_2_-VASc stroke risk score was coded as two or more (which is a commonly used threshold for OAC initiation),^11^ between 1 January 1998 and 30 November 2018, and had at least 1 year of available GP records prior to AF diagnosis (Supplementary material online, *Figure S1*). The study start date was the day that their CHA_2_DS_2_-VASc was coded as two or more.

The primary outcome was a composite of all-cause mortality, ischaemic or unspecified stroke, systemic embolism, major bleeding event that led to hospital admission or death, or any intra-cranial bleeding. Secondary outcomes were all-cause mortality; ischaemic or unspecified stroke; severe bleeding (defined as bleeding that led to hospital admission, death, or any intra-cranial bleeding); TIA; and falls. The date and cause of death was ascertained from linked Office for National Statistics data and was provided as part of the anonymized patient-level dataset. All other outcomes were ascertained from HES and CPRD.

Frailty was ascertained on the study start date using the electronic frailty index (eFI), in which primary care EHR are used to calculate the proportion of deficits (symptoms and signs, abnormal laboratory values, disability, or disease state) from a total of 36 possible deficits. This was then categorized into fit (0–0.12), mild (>0.12–0.24), moderate (>0.24–0.36), or severe (>0.36) frailty.^3^ The eFI is recommended by the National Institute for Health and Care Excellence to identify adults with multimorbidity who are at risk of adverse events. When ICD codes were used to calculate eFI, they were mapped from the originally defined CTV3 codes. With the exception of polypharmacy (≥5 prescriptions in preceding 12-months), deficits were identified if they were recorded at any time point in a patient’s EHR preceding their inclusion.^3^

Baseline characteristics were reported by frailty category, including patient demographics [age, sex, postcode, IMD, ethnicity, smoking status (ever vs. never)], medical history [of stroke or TIA, heart failure, diabetes mellitus, hypertension, peripheral vascular disease (PVD), renal disease, liver disease, previous intra-cranial, or gastrointestinal bleeding). Risk of stroke (CHA_2_DS_2_-VASc) and bleeding [Anticoagulation and Risk Factors in Atrial Fibrillation study (ATRIA): anaemia, severe renal disease, age ≥75 years, prior haemorrhage, hypertension^12^;and modified HAS-BLED score: one point for hypertension, renal or liver disease, stroke, major bleeding or predisposition to bleeding, age >65 years, medication use predisposing to bleeding, or alcohol misuse. Labile INR was omitted as this is not consistently recorded in the dataset] are reported by frailty category.^8,^^13^ The most recent OAC agent prescribed [direct oral anticoagulant (DOAC) or vitamin K antagonist (VKA)], and prescription of the following medications after the index date that may influence the choice to prescribe OAC were reported: antiplatelet medications, proton pump inhibitors (PPI), statins, phenytoin, carbamazepine, macrolide antibiotics, non-steroidal anti-inflammatory drugs (NSAID), and corticosteroids.

### Statistical analyses

Unadjusted rates of the primary and secondary outcomes were reported, alongside those age-standardized to the 2013 European Standard Population. Patients were censored at death, withdrawal from CPRD (e.g. moving to a non-CPRD general practice), or study end (30 November 2018). Fine–Gray competing risk models were used to estimate the hazard ratio (HR) for each outcome with death as a competing risk. After testing assumptions, HRs with 95% confidence intervals (95% CI) for each outcome were reported by frailty status, adjusted for age, sex, IMD, smoking status, CHA_2_DS_2_-VASc score, index year, prescription of aspirin and statin, and comorbidities including diabetes, heart failure, myocardial infarction, hypertension, and PVD. A random intercept for general practice code was included to account for the clustering effect. The prescription of OAC was included as a time-varying variable accounting for the on/off anticoagulation status for each patient throughout the study period. If an OAC prescription was recorded within the 90-days preceding an outcome event, the patient was categorized as being prescribed OAC. Participants were excluded from the main analysis if they died within 3 months of the index date, to allow sufficient time between diagnosis of AF to allow OAC to be commenced. Cumulative incidence functions were visualized for each clinical outcome, stratified by frailty category and time-varying OAC prescription. Age-standardized incidences were calculated according to European Standard Population by frailty category and OAC prescription, and adjusted to duration of follow-up to account for the differing length of follow-up for DOAC and VKA. Data were collected on a positive recording basis, whereby the absence of a recorded diagnosis is treated as the absence of that event. Therefore, no formal missing data strategy was employed. Analyses were undertaken using R (version 3.6.3) with statistical significance determined at *P* < 0.05.

### Role of the funder

The funder had no role in the study design; in the collection, analysis, and interpretation of data; in the writing of the report; or in the decision to submit the article for publication. The researchers are independent of the funders.

### Ethics

The protocol for CPRD has been approved by the Independent Scientific Advisory Committee for MHRA Database Research. This study was conducted in accordance with the Declaration of Helsinki and is reported in line with RECORD recommendations. J.W. had full access to the data and can take responsibility for the integrity of the data and the accuracy of the data analysis. All authors take responsibility for the interpretation of the analyses.

## Results

The cohort comprised 89 996 participants. In total, 18 740 (20.8%) were fit and 71 256 (79.2%) were living with frailty (mild: 33 674, moderate 25 686, severe 11 896, *Table 1*). The mean age of participants was 78.3 (SD 9.5, range 18–108) years and 45.5% were male. There were 369 489 person-years of follow-up (median 2.8, IQR 1.2–5.5 years).

**Table 1:** Characteristics of participants by frailty status at study entry

	All	Fit	Mild frailty	Moderate frailty		Severe frailty
	89 996	18 740	33 674	25 686		11 896
Demographics, *n* (%)						
Age, mean (SD)	78.33 (9.50)	76.61 (10.04)	77.63 (9.77)	79.53 (8.89)		80.44 (8.35)
Male	40 950 (45.5)	8714 (46.5)	16 389 (48.7)	11 343 (44.2)		4504 (37.9)
IMD						
1	19 500 (21.7)	4451 (23.8)	7498 (22.3)	5276 (20.5)		2275 (19.1)
2	19 345 (21.5)	4238 (22.6)	7466 (22.2)	5421 (21.1)		2220 (18.7)
3	20 393 (22.7)	4198 (22.4)	7632 (22.7)	5818 (22.7)		2745 (23.1)
4	17 000 (18.9)	3380 (18.1)	6244 (18.6)	4977 (19.4)		2399 (20.2)
5	13 705 (15.2)	2452 (13.1)	4812 (14.3)	4187 (16.3)		2254 (19.0)
Ethnicity, white	84 382 (94.9)	17 032 (93.0)	31 363 (94.5)	24 485 (95.8)		11 502 (96.9)
Ever smoked	44 203 (54.3)	7303 (47.8)	16 791 (54.6)	13 666 (56.9)		6443 (56.9)
Medical history						
Previous stroke/TIA	12 448 (13.8)	944 (5.0)	4098 (12.2)	4483 (17.5)		2923 (24.6)
Previous stroke	6779 (7.5)	526 (2.8)	2255 (6.7)	2414 (9.4)		1584 (13.3)
Previous TIA	7283 (8.1)	502 (2.7)	2249 (6.7)	2665 (10.4)		1867 (15.7)
Previous MI	10 500 (11.7)	889 (4.7)	3332 (9.9)	3810 (14.8)		2469 (20.8)
Heart failure	10 899 (12.1)	542 (2.9)	3158 (9.4)	4203 (16.4)		2996 (25.2)
Diabetes	16 842 (18.7)	1795 (9.6)	5502 (16.3)	5758 (22.4)		3787 (31.8)
Hypertension	54 914 (61.0)	7841 (41.8)	20 214 (60.0)	17 748 (69.1)		9111 (76.6)
PVD	4353 (4.8)	143 (0.8)	1031 (3.1)	1727 (6.7)		1452 (12.2)
Renal disease	16 923 (18.8)	728 (3.9)	5365 (15.9)	6651 (25.9)		4179 (35.1)
Liver disease	283 (0.3)	34 (0.2)	101 (0.3)	111 (0.4)		37 (0.3)
Previous major bleeding						
Intra-cranial	72 (0.1)	10 (0.1)	18 (0.1)	21 (0.1)		23 (0.2)
Gastrointestinal	8939 (9.9)	879 (4.7)	2906 (8.6)	3161 (12.3)		1993 (16.8)
CHA_2_DS_2_-VASc						
2	26 487 (29.4)	8863 (47.3)	10 869 (32.3)	5267 (20.5)		1488 (12.5)
3	30 531 (33.9)	7037 (37.6)	12 192 (36.2)	8235 (32.1)		3067 (25.8)
4	24 034 (26.7)	2525 (13.5)	8542 (25.4)	8641 (33.6)		4326 (36.4)
5	7109 (7.9)	292 (1.6)	1779 (5.3)	2834 (11.0)		2204 (18.5)
6	1520 (1.7)	21 (0.1)	259 (0.8)	599 (2.3)		641 (5.4)
7	267 (0.3)	2 (0.0)	28 (0.1)	102 (0.4)		135 (1.1)
8	45 (0.1)	0 (0.0)	5 (0.0)	8 (0.0)		32 (0.3)
9	3 (0.0)	0 (0.0)	0 (0.0)	0 (0.0)		3 (0.0)
ATRIA score						
<4—low risk	61 727 (68.6)	17 003 (90.7)	24 531 (72.8)	14 969 (58.3)		5224 (43.9)
4—medium risk	4263 (4.7)	446 (2.4)	1826 (5.4)	1381 (5.4)		610 (5.1)
>4—high risk	24 006 (26.7)	1291 (6.9)	7317 (21.7)	9336 (36.3)		6062 (51.0)
Modified HAS-BLED, mean (SD)	2.73 (0.99)	2.17 (0.83)	2.65 (0.92)	2.98 (0.95)		3.29 (0.98)
Medications						
Oral anticoagulation						
Any OAC	43 228 (48.0)	5053 (27.0)	16 603 (49.3)	14 293 (55.6)		7256 (61.0)
DOAC	10 352 (11.5)	1382 (7.4)	3967 (11.8)	3258 (12.7)		1745 (14.7)
Apixaban	4558 (5.1)	580 (3.1)	1722 (5.1)	1472 (5.7)		784 (6.6)
Dabigatran	1122 (1.2)	157 (0.8)	437 (1.3)	341 (1.3)		187 (1.6)
Edoxaban	415 (0.5)	53 (0.3)	182 (0.5)	130 (0.5)		50 (0.4)
Rivaroxaban	5164 (5.7)	677 (3.6)	1932 (5.7)	1620 (6.3)		935 (7.9)
VKA	32 876 (36.5)	3671 (19.6)	12 636 (37.5)	11 058 (43.1)		5511 (46.3)
Warfarin	32 809 (36.5)	3660 (19.5)	12 613 (37.5)	11 035 (43.0)		5501 (46.2)
Acenocoumarol	168 (0.2)	19 (0.1)	55 (0.2)	58 (0.2)		36 (0.3)
Phenindione	57 (0.1)	4 (0.0)	22 (0.1)	20 (0.1)		11 (0.1)
Antiplatelet prescription at any time during follow-up						
Aspirin	43 034 (47.8)	4554 (24.3)	15 973 (47.4)	14 855 (57.8)		7652 (64.3)
Clopidogrel	10 547 (11.7)	629 (3.4)	3208 (9.5)	3997 (15.6)		2713 (22.8)
Prasugrel	47 (0.1)	3 (0.0)	19 (0.1)	16 (0.1)		9 (0.1)
Ticagrelor	162 (0.2)	20 (0.1)	59 (0.2)	48 (0.2)		35 (0.3)
Dipyridamole	2692 (3.0)	155 (0.8)	793 (2.4)	1054 (4.1)		690 (5.8)
Other medication at any time during follow-up						
PPI	40 884 (45.4)	3493 (18.6)	14 371 (42.7)	14 740 (57.4)		8280 (69.6)
Statin	40 779 (45.3)	3876 (20.7)	15 126 (44.9)	14 215 (55.3)		7562 (63.6)
Phenytoin	477 (0.5)	51 (0.3)	147 (0.4)	185 (0.7)		94 (0.8)
Carbamazepine	1101 (1.2)	67 (0.4)	345 (1.0)	425 (1.7)		264 (2.2)
Macrolide antibiotics	17 411 (19.3)	1084 (5.8)	5370 (15.9)	6618 (25.8)		4339 (36.5)
NSAIDS	16 577 (18.4)	1342 (7.2)	5660 (16.8)	5974 (23.3)		3601 (30.3)
Corticosteroids	46 895 (52.1)	4085 (21.8)	16 880 (50.1)	16 853 (65.6)		9077 (76.3)

ATRIA, one point each for anaemia, severe renal disease, prior haemorrhage, or hypertension. Two points for age ≥75 years. Three points for severe renal disease; CHA_2_DS_2_-VASc, one point for age 65–74 years, female sex; history of heart failure, hypertension, vascular disease, or diabetes. Two points are allocated for age >75 years, and two points for a history of stroke, transient ischaemic attack, or thromboembolism; DOAC, Direct Oral Anticoagulant; modified HAS-BLED, one point for hypertension, renal or liver disease, stroke, major bleeding or predisposition to bleeding, age >65 years, medication use predisposing to bleeding or alcohol misuse; MI, myocardial infarction; NSAID, non-steroidal anti-inflammatory drug; PPI, proton pump inhibitor; SD, standard deviation; VKA, vitamin K antagonist.

With increasing frailty category, participants tended to be older (fit: 76.6, severe frailty: 80.4 years), were more commonly women (fit: 53.5%, severe frailty: 62.1%) and with a history of smoking (fit: 47.8%, severe frailty: 56.9%). The proportion of participants with a history of gastrointestinal bleeding was higher with increasing frailty category (fit: 4.7%, severe frailty: 16.8%) and people living with frailty tended to have higher CHA_2_DS_2_-VASc and ATRIA scores (*Table 1*).

Overall, 43 228 (48.0%) participants were prescribed OAC during their analytical period. Of these, DOAC was prescribed in 23.9% and VKA in 76.1%. Prescription rates of OAC were higher in patients with increasing frailty (fit: 27.0%, mild frailty: 49.3%, moderate: 55.6%, severe: 61.0%). Prescription rates of anti-platelet medication were also higher with increasing frailty (aspirin: fit 24.3%, severe frailty 64.3%; clopidogrel: fit 3.4%; severe frailty 22.8%), although this was not necessarily concomitant with OAC.

### Composite clinical outcomes—standardized to the European populace

The composite clinical outcome occurred in 48 311 (53.7%) people (Supplementary material online, *Table S1*). Overall, the prescription of OAC was associated with a reduction in the rates of the composite clinical endpoint. For patients who were not prescribed OAC, the incidence rates (IR, per 100 person-years) of the composite outcomes increased with increasing frailty category [fit: IR 4.8, 95% CI 4.7–4.8; mild frailty: IR 5.9, 95% CI 5.8–6.0; moderate: IR 6.8, 95% CI 6.6–6.9; severe: IR 8.7, 95% CI 8.3–9.0 (*Table 2*); crude rates are shown in Supplementary material online, *Table S2*]. However, in those prescribed VKA, the IR of composite outcomes did not increase consistently with increasing frailty (fit: IR 4.3, 95% CI 3.7–4.9; mild frailty: IR 7.3, 95% CI 6.9–7.8; moderate: IR 5.6, 95% CI 5.4–5.8; severe: IR 8.6, 95% CI 8.1–9.0). In those prescribed DOAC, event rates were lower than those prescribed VKA and those not prescribed OAC in all but the severe frailty category (fit: IR 0.9, 95% CI 0.8–1.0; mild frailty: IR 1.8, 95% CI 1.7–1.9; moderate: IR 1.7, 95% CI 1.6–1.8; severe: 9.5, 95% CI 8.7–10.4).

**Table 2:** Age-standardized incidence rate per 100 person-years for composite and secondary outcomes, by frailty status and OAC prescription

	Incidence rate per 100 person-years (95% confidence interval)											
	Fit			Mild frailty			Moderate frailty			Severe frailty		
Outcome	No OAC	VKA	DOAC	No OAC	VKA	DOAC	No OAC	VKA	DOAC	No OAC	VKA	DOAC
Composite	4.8 (4.7–4.8)	4.3 (3.7–4.9)	0.9 (0.8–1.0)	5.9 (5.8–6.0)	7.3 (6.9–7.8)	1.8 (1.7–1.9)	6.8 (6.6–6.9)	5.6 (5.4–5.8)	1.7 (1.6–1.8)	8.7 (8.3–9.0)	8.6 (8.1–9.0)	9.5 (8.7–10.4)
Death	3.9 (3.9–4.0)	3.8 (3.2–4.3)	0.7 (0.6–0.8)	4.7 (4.6–4.8)	5.0 (4.7–5.4)	1.1 (1.0–1.2)	5.5 (5.4–5.6)	3.3 (3.2–3.5)	1.3 (1.2–1.4)	4.6 (4.5–4.6)	4.9 (4.7–5.1)	8.4 (7.6–9.3)
Ischaemic stroke	0.4 (0.4–0.4)	0.4 (0.4–0.5)	0.1 (0.1–0.1)	0.6 (0.6–0.6)	0.3 (0.3–0.4)	0.4 (0.4–0.4)	0.8 (0.7–0.8)	0.6 (0.5–0.6)	0.1 (0.1–0.2)	1.0 (0.9–1.0)	0.6 (0.6–0.6)	0.4 (0.3–0.4)
All stroke	0.5 (0.5–0.5)	0.7 (0.6–0.7)	0.1 (0.1–0.1)	0.6 (0.6–0.7)	0.5 (0.5–0.6)	0.5 (0.4–0.5)	0.8 (0.8–0.9)	0.7 (0.7–0.7)	0.2 (0.1–0.3)	1.0 (1.0–1.0)	0.7 (0.7–0.8)	0.4 (0.4–0.4)
Severe bleeding	0.8 (0.8–0.8)	0.6 (0.6–0.6)	0.1 (0.1–0.1)	0.7 (0.7–0.8)	2.5 (2.2–2.8)	0.4 (0.4–0.4)	0.7 (0.7–0.8)	2.1 (2.0–2.2)	0.2 (0.2–0.3)	3.2 (2.9–3.6)	1.3 (1.3–1.3)	0.7 (0.7–0.7)
TIA	0.1 (0.1–0.1)	0.1 (0.1–0.2)	0.0 (0.0–0.0)	0.2 (0.2–0.2)	0.1 (0.1–0.1)	0.1 (0.1–0.1)	0.3 (0.3–0.3)	0.5 (0.5–0.5)	0.1 (0.1–0.1)	0.2 (0.2–0.2)	0.3 (0.3–0.4)	0.3 (0.2–0.3)
Fall	0.3 (0.3–0.4)	0.4 (0.0–1.0)	0.1 (0.1–0.1)	0.6 (0.6–0.6)	1.6 (1.4–1.8)	2.7 (2.3–3.0)	1.8 (1.7–1.8)	2.0 (1.9–2.1)	0.4 (0.4–0.4)	3.2 (3.2–3.3)	3.2 (3.0–3.4)	0.9 (0.8–0.9)

DOAC, direct oral anticoagulant; OAC, oral anticoagulant; TIA, transient ischaemic attack; VKA, vitamin K antagonist.

### Composite clinical outcomes—on treatment and adjusted

**Figure 1:**
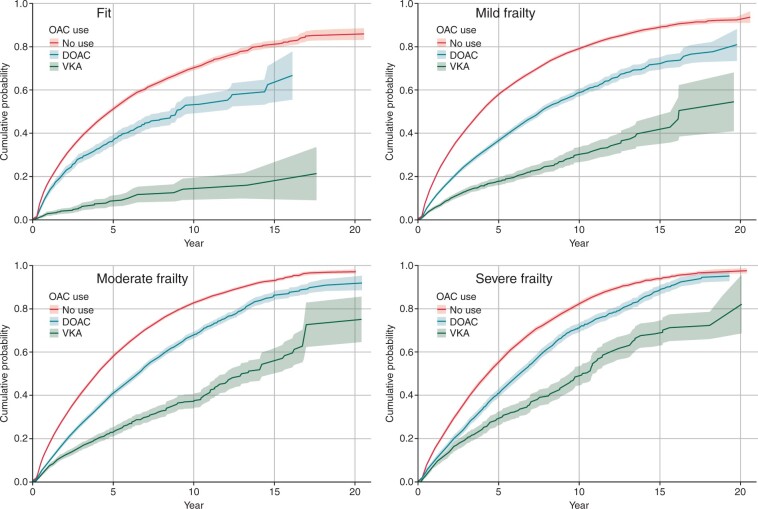
Cumulative incidence function for composite outcome (death, stroke, systemic embolism, gastrointestinal, or intra-cranial haemorrhage) by frailty category and time-varying anticoagulation status (with 95% confidence intervals).

The cumulative incidence function shows that prescription of OAC was associated with a substantial reduction in the composite clinical outcome (*Figure 1*).

In models further adjusted for demographics, stroke risk, other medications, cardiovascular comorbidities, and accounting for OAC as a time-varying covariate, the prescription of DOAC, or VKA were associated with a consistent reduction in composite clinical outcomes across all frailty categories compared with no OAC (*Table 3*). VKA was associated with an average reduction in the composite endpoint of 31% in the fit group (HR 0.69, 95% CI 0.64–0.75), 48% in those with mild frailty (HR 0.52, 95% CI 0.50–0.54), 46% in those with moderate frailty (HR 0.54, 95% CI 0.52–0.56), and 52% in those with severe frailty (HR 0.48, 95% CI 0.45–0.51). DOAC was associated with an average reduction of 58% in the fit group (HR 0.42, 95% CI 0.33–0.53), 43% in those with mild frailty (HR 0.57, 95% CI 0.52–0.63), 43% with moderate frailty (HR 0.57, 95% CI 0.52–0.63), and 42% with severe frailty (HR 0.58, 95% CI 0.52–0.65).

**Table 3:** The association between oral anticoagulation and outcomes, stratified by frailty category

	Adjusted hazard ratio (95% confidence interval) compared to no anticoagulation (reference), within each frailty category							
	Fit		Mild frailty		Moderate frailty		Severe frailty	
	VKA	DOAC	VKA	DOAC	VKA	DOAC	VKA	DOAC
Composite	0.69 (0.64–0.75)	0.42 (0.33–0.53)	0.52 (0.50–0.54)	0.57 (0.52–0.63)	0.54 (0.52–0.56)	0.57 (0.52–0.63)	0.48 (0.45–0.51)	0.58 (0.52–0.65)
Death	0.70 (0.64–0.76)	0.41 (0.31–0.53)	0.48 (0.46–0.50)	0.52 (0.47–0.58)	0.47 (0.45–0.49)	0.57 (0.52–0.62)	0.39 (0.37–0.42)	0.55 (0.49–0.61)
Ischaemic stroke	0.46 (0.35–0.61)	0.49 (0.25–0.95)	0.44 (0.39–0.50)	0.58 (0.43–0.77)	0.57 (0.51–0.63)	0.43 (0.32–0.59)	0.50 (0.43–0.58)	0.54 (0.39–0.75)
All stroke	0.70 (0.57–0.86)	0.60 (0.35–1.03)	0.58 (0.52–0.64)	0.66 (0.52–0.84)	0.59 (0.53–0.65)	0.47 (0.36–0.61)	0.53 (0.47–0.61)	0.52 (0.39–0.70)
Severe bleeding	0.91 (0.74–1.11)	0.43 (0.24–0.77)	0.94 (0.85–1.04)	1.07 (0.87–1.32)	1.06 (0.97–1.17)	0.88 (0.71–1.10)	1.00 (0.88–1.13)	1.24 (0.97–1.57)
TIA	0.43 (0.23–0.79)	0.32 (0.08–1.31)	0.59 (0.46–0.77)	0.51 (0.28–0.93)	0.62 (0.50–0.77)	0.80 (0.52–1.24)	0.71 (0.55–0.92)	0.65 (0.37–1.13)
Fall	2.53 (1.87–3.43)	2.24 (1.06–4.76)	1.49 (1.36–1.64)	1.36 (1.08–1.70)	1.19 (1.11–1.28)	1.21 (1.02–1.43)	1.24 (1.14–1.34)	1.28 (1.06–1.53)

Each model was performed by frailty status adjusted for age, sex, deprivation index, smoking, CHA_2_DS_2_-VASc score, medication on aspirin and statin, comorbidities including history of diabetes, heart failure, myocardial infarction, hypertension, peripheral vascular disease, and index year. A random intercept for practices was included to account for the clustering effect. The prescription of OAC (including VKA and DOAC) was included as time-varying variables accounting for the on/off anticoagulation status for each patient.

DOAC, direct oral anticoagulant; OAC, oral anticoagulant; TIA, transient ischaemic attack; VKA, vitamin K antagonist.

### Secondary clinical outcomes

#### All-cause mortality

**Figure 2:**
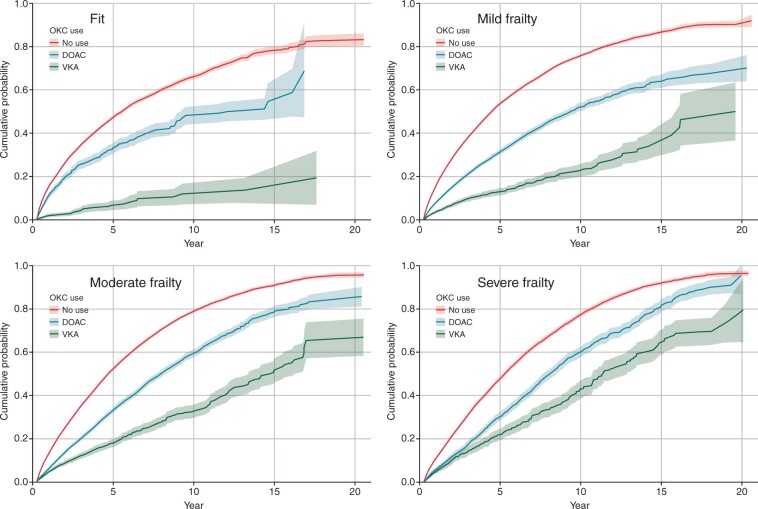
Cumulative incidence function for all-cause death by frailty category and time-varying anticoagulation status (with 95% confidence intervals).

There were 44 380 (49.3%) deaths during the follow-up period. *Figure 2* shows that for each frailty category, mortality rates were lowest amongst patients prescribed DOAC, then VKA and highest amongst people who were not prescribed OAC (*Figure 2*). Standardized mortality rates were higher with increasing frailty compared with those who were fit (*Table 2*) and were lowest for those that were prescribed DOAC in the fit, mild, and moderate frailty groups. In the group with severe frailty, those prescribed DOAC had a higher rate of mortality (IR 8.4, 95% CI 7.6–9.3) than those prescribed VKA and those not prescribed OAC (VKA, IR 4.9, 95% CI 4.7–5.1; no OAC, IR 4.6, 95% CI 4.5–4.6).

The adjusted analyses show that OAC prescription was associated with a reduction in mortality across all four categories compared with no OAC prescription (HR for VKA vs. no OAC: fit 0.70, 95% CI 0.64–0.76; mild frailty 0.48, 95% CI 0.46–0.50; moderate 0.47, 95% CI 0.45–0.49; severe 0.39, 95% CI 0.37–0.42. Hazard ratio for DOAC vs. no OAC: Fit 0.41, 95% CI 0.31–0.53; mild frailty 0.52, 95% CI 0.47–0.58; moderate 0.57, 95% CI 0.52–0.62; severe 0.55, 95% CI 0.49–0.61).

#### Stroke

**Figure 3:**
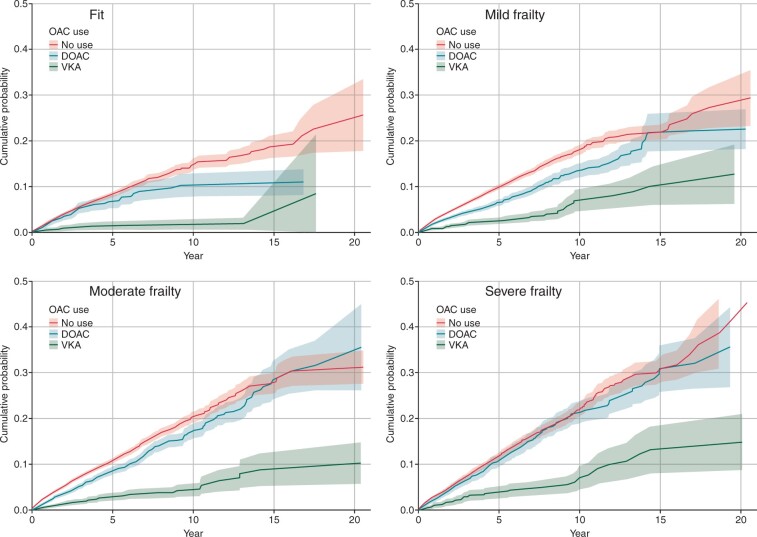
Cumulative incidence function for stroke by frailty category and anticoagulation status (with 95% confidence intervals).

Overall, 7028 (7.8%) participants had a stroke during follow-up, 84.0% (*n* = 5896) of which were ischaemic. Prescription of DOAC was associated with a substantially lower risk of stroke than VKA or no OAC prescription (*Figure 3*). Standardized rates tended to be higher with increasing frailty category, and lower in those that were prescribed OAC—but without a consistent benefit of one agent over the other across the frailty categories (*Table 2*). Following adjustment, prescription of VKA or DOAC was associated with a reduction in ischaemic stroke across every frailty category compared with no OAC (HR for VKA vs. no OAC: fit 0.46, 95% CI 0.35–0.61; mild frailty 0.44, 0.39–0.50; moderate 0.57, 0.51–0.63; severe 0.50, 0.43–0.58. The HR for DOAC vs. no OAC: fit 0.49, 0.25–0.95; mild frailty 0.58, 0.43–0.77; moderate 0.43, 0.32–0.59; severe 0.54, 0.39–0.75).

#### Severe bleeding

**Figure 4:**
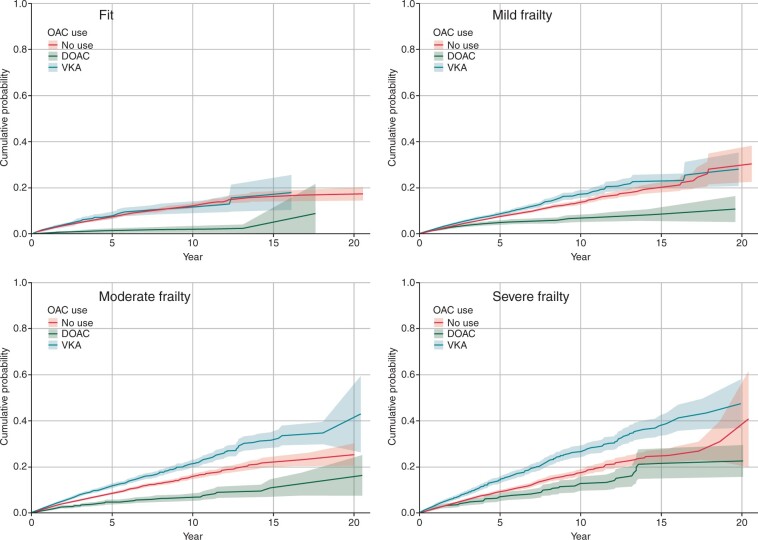
Cumulative incidence function for severe bleeding by frailty category and anticoagulation status (with 95% confidence intervals).

Severe bleeding occurred in 6401 (7.1%) people and was more frequent with increasing frailty (*Figure 4*). The standardized rates of bleeding showed no consistent pattern between agents across the frailty categories (*Table 2*), whereas the adjusted models showed that OAC prescription was associated with a similar bleeding risk than no OAC—except for in the fit group prescribed DOAC, in whom bleeding appeared less common than no OAC (HR for VKA vs. no OAC: fit 0.91, 95% CI 0.74–1.11; mild frailty 0.94, 95% CI 0.85–1.04; moderate 1.06, 95% CI 0.97–1.17; severe 1.00, 95% CI 0.88–1.13; HR for DOAC vs. no OAC, fit 0.43, 95% CI 0.24–0.77; mild frailty 1.07, 95% CI 0.87–1.32; moderate 0.88, 95% CI 0.71–1.10; severe 1.24, 95% CI 0.97–1.57, *Table 3*).

#### Transient ischaemic attack

**Figure 5:**
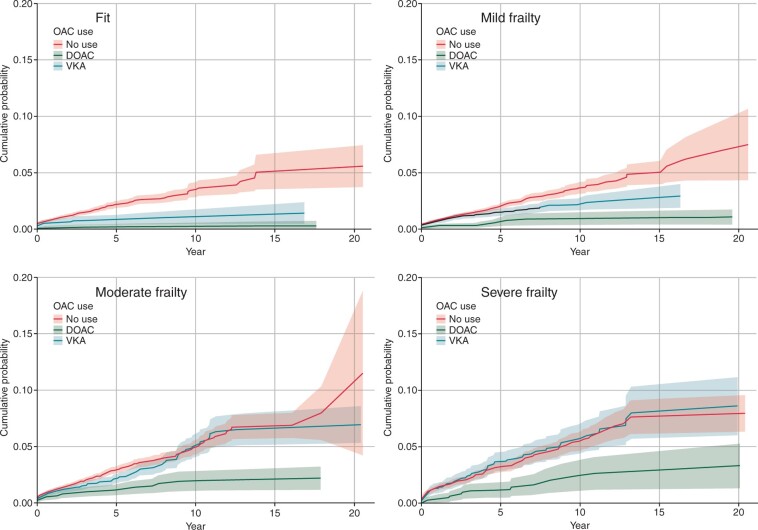
Cumulative incidence function for transient ischaemic attack by frailty category and anticoagulation status.

There were 1785 (2.0%) TIAs, with the lowest event rates observed in people prescribed DOAC (*Figure 5*). Standardized rates increased with frailty (*Table 2*). Following adjustment, the prescription of VKA was associated with a consistent reduction in TIA rate across all frailty categories (HR for VKA vs. no OAC: fit 0.43, 95% CI 0.23–0.79; mild frailty 0.59, 95% CI 0.46–0.77; moderate 0.62, 95% CI 0.50–0.77; severe 0.71, 95% CI 0.55–0.92), but the reduction with DOAC was only statistically significant in the group with mild frailty (HR for DOAC vs. no OAC: fit 0.32, 95% CI 0.08–1.31; mild frailty 0.51, 95% CI 0.28–0.93; moderate 0.80, 95% CI 0.52–1.24; severe 0.65, 95% CI 0.37–1.13, *Table 3*).

#### Falls

Overall, 9931 (11.0%) participants had a fall recorded. Falls were more common with increasing frailty and tended to occur more frequently in patients prescribed VKA than no OAC or DOAC (Supplementary material online, *Figure S2*). In the adjusted analyses, on average, those prescribed OAC more commonly had a fall than those not prescribed OAC (HR for VKA vs. no OAC: fit 2.53, 95% CI 1.87–3.43; mild frailty 1.49, 95% CI 1.36–1.64; moderate 1.19, 95% CI 1.11–1.28; severe 1.24, 95%CI 1.14–1.34. Hazard ratio for DOAC vs. no OAC: fit 2.24, 95% CI 1.06–4.76; mild frailty 1.36, 95% CI 1.08–1.70; moderate 1.21, 95% CI 1.02–1.43; severe 1.28, 95% CI 1.06–1.53, *Table 3*).

## Discussion

This cohort study included 89 996 participants and used primary care EHR linked to hospital and mortality data to study the on-treatment effects of OAC on clinical outcomes among people with AF according to frailty status. We found that frailty was more commonly associated with adverse clinical outcomes in patients with AF and, although the use of OAC for stroke prophylaxis increased with increasing frailty category, overall the use of OAC was suboptimal. Moreover, we found that the prescription of OAC was associated with a substantial reduction in the composite endpoint of death, stroke, systemic embolism, and major bleeding across the frailty spectrum.

The study benefitted from a large sample size, a long duration of follow-up, and addresses a topical and important clinical issue. We used a robust, validated and guideline-recommended measure of frailty, and a linked dataset for outcome ascertainment. Nonetheless, we recognize the limitations of our work. We were reliant on the accurate identification and coding of events in a routine dataset, which may not be completely accurate.^14^ There have been changes in clinical guidance over the duration of the study follow-up period. Nevertheless, the thresholds used for this study are based upon current UK guidance, and so are applicable to contemporary practice.^11^ As we lacked data on treatment adherence, prescription of OAC does not necessarily mean that it was taken, thereby possibly under-estimating strength of association.^15^ We estimated frailty when the patient became eligible for prescription of OAC, as this is the key inflection point for clinical decision making, however, frailty is a dynamic phenomenon and patients are likely to have accumulated further deficits over the follow-up period,^3^ and coding practices may have changed over time. There was a small difference in the duration of follow-up between groups, although this was accounted for in the primary analysis by standardization and fitting time-varying exposure of OAC prescription. Although adjustment was made for potential confounders, there is likely to be residual unmeasured confounding including confounding by indication. Finally, this was an observational study; therefore, we describe associations and cannot attribute causation or a comparison between treatments.

In the original trials of stroke prophylaxis in AF, each DOAC agent was compared with VKA. Meta-analysis of these trials has shown that overall, DOACs have favourable efficacy and safety profiles compared with warfarin.^16^ In a subgroup meta-analysis of older people, there was superior stroke prevention in the DOAC group than the VKA group, and whilst the intra-cranial haemorrhage rate was lower in patients randomized to a DOAC the overall rate of major bleeding was similar between the two groups.^17^

Our finding that there was a greater reduction in the risk of the composite outcome with VKA compared with DOAC in people with mild, moderate, and severe frailty is of interest. Whilst a head-to-head comparison of treatments is not possible in this observational study, this is an important avenue for future work. There are no randomized clinical trials comparing DOAC and VKA specifically for a population with frailty, and of those trials comparing DOAC and VKA the proportion of participants who were frail was limited. For example, only one-fifth of the people recruited into the ENGAGE AF-TIMI 48 trial were living with frailty^18^; this compares with almost four-fifths in this real-world naturalistic study. The recent *post-hoc* analysis of the ENGAGE AF-TIMI 48 trial showed similar efficacy to warfarin across the frailty spectrum, with lower rates of bleeding except in those with severe frailty.^18^ Furthermore, observational work suggests that there may be differences in the efficacy and safety between DOAC agents for different degrees of frailty.^19^ Although there is a need for randomized evidence to evaluate the safety of efficacy of DOAC compared with VKA in people with frailty, we recognize that a comparative effectiveness trial is unlikely given that conducting a trial in this population may be challenging.

The population burden of AF is growing, as is the proportion of people with AF that are also living with frailty. We have shown that this group of people have poor clinical outcomes, especially if they are not prescribed OAC. Over the 20 year period we found that OAC prescription rates were low, but this will likely be a reflection of the temporal increase of the use of OAC in the UK.^20^ Moreover, we found a positive association between frailty and OAC prescription, which validates previous findings, and may reflect that practitioners are considering the high risk of stroke in people with advancing frailty. Even so, we also show that the risk of severe bleeding is highest in people with frailty, as is the rate of falls. These findings reinforce the importance of minimizing bleeding risk through reviewing concomitant therapy associated with bleeding such as NSAIDs and anti-platelet medications,^11^ and adopting a multi-disciplinary approach to mitigating falls risk.

## Conclusion

In this large, community-based cohort study of people with AF, frailty was associated with adverse clinical outcomes in patients with AF. However, OAC prescription was associated with substantial reductions in the composite endpoint of death, stroke, systemic embolism, and major bleeding across the frailty spectrum.

## Supplementary material

Supplementary material is available at *Europace* online.

## Supplementary Material

euac022_Supplementary_Data
